# A Review of Nutraceuticals in Cancer Cachexia

**DOI:** 10.3390/cancers15153884

**Published:** 2023-07-30

**Authors:** Lucas Caeiro, Devika Gandhay, Lindsey J. Anderson, Jose M. Garcia

**Affiliations:** 1 Geriatric Research, Education and Clinical Center, Veterans Affairs Puget Sound Health Care System, Seattle, WA 98108, USAlindsey.anderson5@va.gov (L.J.A.); 2 Division of Gerontology and Geriatric Medicine, Department of Medicine, University of Washington School of Medicine, Seattle, WA 98195, USA

**Keywords:** nutraceuticals, cancer cachexia, physical function, muscle mass, quality of life, handgrip strength, patient-reported outcomes

## Abstract

**Simple Summary:**

Cancer-related muscle wasting and inflammation, known as cachexia, leads to weight loss and worsened physical function, quality of life (QOL), and survival. The main barrier to current treatments is the lack of improvement in clinically relevant outcomes (function, QOL). Nutraceuticals are naturally occurring food products, which may be of benefit in cancer cachexia. This review describes the effect of nutraceuticals in animal models and in clinical trials in patients with cancer cachexia. Human studies mostly tested fish oil (or something similar) or amino acids (the building blocks of proteins). Body weight was the main focus, while some also assessed muscle mass and QOL, and very few measured physical function. The safety and efficacy of nutraceuticals in treating cancer-related muscle wasting remains uncertain. More animal and large human studies are needed, and they should focus on clinically meaningful outcomes, such as physical function and QOL.

**Abstract:**

Cancer cachexia is largely characterized by muscle wasting and inflammation, leading to weight loss, functional impairment, poor quality of life (QOL), and reduced survival. The main barrier to therapeutic development is a lack of efficacy for improving clinically relevant outcomes, such as physical function or QOL, yet most nutraceutical studies focus on body weight. This review describes clinical and pre-clinical nutraceutical studies outside the context of complex nutritional and/or multimodal interventions, in the setting of cancer cachexia, in view of considerations for future clinical trial design. Clinical studies mostly utilized polyunsaturated fatty acids or amino acids/derivatives, and they primarily focused on body weight and, secondarily, on muscle mass and/or QOL. The few studies that measured physical function almost exclusively utilized handgrip strength with, predominantly, no time and/or group effect. Preclinical studies focused mainly on amino acids/derivatives and polyphenols, assessing body weight, muscle mass, and occasionally physical function. While this review does not provide sufficient evidence of the efficacy of nutraceuticals for cancer cachexia, more preclinical and adequately powered clinical studies are needed, and they should focus on clinically meaningful outcomes, including physical function and QOL.

## 1. Introduction

Cancer cachexia is a complex metabolic syndrome characterized by loss of muscle—with or without loss of fat mass—that is not reversed by conventional nutritional supplementation and leads to progressive functional impairment [1]. Factors such as inflammation and insulin resistance drive negative protein/energy balance, and they lead to poorer quality of life (QOL), as well as declines in physical function [2,3,4]. The operational definition of cachexia is unintentional weight loss and/or low body mass index (BMI) or muscle mass (>5% weight loss over six months, BMI < 20 kg/m^2^ with >2% weight loss, or sarcopenia with >2% weight loss) [1]. Cachexia is present in up to 80% of patients with cancer, and it is associated with up to 30% of cancer-related deaths [5], yet there are no treatments currently approved for this indication by the U.S. Food and Drug Administration or the European Medicines Agency. This is primarily due to the lack of clinically meaningful improvements in physical function reported by phase III clinical trials to date, despite that many improved muscles mass [6,7,8,9,10]. However, muscle mass, unlike physical function and QOL, is not considered a clinically relevant outcome, and novel interventions to improve functional performance and QOL are needed to advance therapeutic development.

Multimodal interventions, including exercise and individualized nutrition, are thought to have the most potential for mitigating cachexia [11], but effective strategies for improving functional performance and QOL have yet to be identified. Natural food/herbal medicine products, loosely termed nutraceuticals, display anti-inflammatory, antioxidant, and anti-cancer properties, making them promising adjuvant treatments in the setting of cancer cachexia [12]. Additionally, some plant-based food products have shown favorable effects on physical function in healthy older adults [13]. However, the primary outcome of most nutraceutical studies in the broader cancer setting is body weight, with few studies including a measure of muscularity, patient-reported (PR-)QOL, and/or subjective physical function, as reviewed elsewhere [14,15,16]. Even fewer studies report objectively measured physical function, but as phase III cancer cachexia trials have shown, increased body weight and muscle mass are often not associated with improved PR-QOL or physical function [6,7,8,9,10].

There is no consensus on the most clinically important functional outcome(s) that should be measured in the setting of cancer cachexia. This review set out to describe the efficacy of nutraceuticals for improving cachexia outcomes such as body weight and muscle mass while highlighting clinically relevant outcomes, including physical function, measured subjectively or objectively, and QOL, in view of considerations for future clinical trial design. In contrast to recently published reviews examining nutraceuticals in the context of exercise [17] or restricted to preclinical data [18], the current review focuses on published clinical trials and animal studies, evaluating the effect of nutraceutical interventions on clinically relevant outcomes in the setting of cancer cachexia. Clinical studies mostly utilized polyunsaturated fatty acids (PUFA; alone or with anti-inflammatories) or amino acids/derivatives, and they primarily focused on body weight and, secondarily, on muscle mass, QOL, and/or physical function, reporting minimal to no improvement. Preclinical studies mainly focused on polyphenols or amino acids, mainly assessing body weight along with muscle mass and, occasionally, physical function. The potential for nutraceuticals to benefit body weight, muscle mass, physical function, and QOL in cancer cachexia remains unclear in humans; animal studies, in general, seem to show more positive results. More preclinical and adequately powered clinical studies are needed, and they should focus on clinically meaningful outcomes, such as physical function and QOL.

## 2. Materials and Methods

The databases used to search were Google Scholar, PubMed, PubMed Central, and ClinicalTrials.gov (last accession date: 17 January 2023 for ClinicalTrials.gov). The search was limited to studies published in English between 1996 and 2023 (July). Search terms included: nutraceutical + one of the following [cancer cachexia, muscle wasting, muscle mass, physical function, body weight, PR-QOL]; cachexia + one of the following [eicosapentaenoic acid (EPA), β-hydroxy β-methylbutyrate (HMB), fatty acids, protein, probiotics, amino acids, antioxidants]. This resulted in 182 publications being retrieved, with 46 papers discarded for being reviews and 78 original articles discarded due to the inclusion and exclusion criteria. This left a total of 37 preclinical and 21 clinical studies. Animal studies had to assess muscularity and/or physical function in addition to body weight (Table 1); clinical trials were required to include at least two of the following outcomes: body weight, muscularity, physical function, or PR-QOL (Table 2). The following study designs were excluded: (1) highly complex nutritional or multimodal interventions, (2) unrelated to cancer cachexia, and (3) in vitro studies.

## 3. Results

### 3.1. Amino Acids and Metabolites

Amino acids are the primary constituents of proteins, are critical for modulation of many cell signals, and are known to promote growth of skeletal muscle [79]. We identified 14 animal studies that administered amino acids to tumor-bearing mice via gavage, enriched diet, or subcutaneous injection. All studies assessed body weight and muscle mass, with 8 reporting body weight improvement and 11 reporting attenuation of muscle wasting. There were four studies that assessed physical function through grip strength, latency to fall, and/or physical activity [24,27,29,31]. All four studies reported improvement in physical function and muscle mass, but only two reported a concomitant improvement in body weight (Table 1) [24,31].

Among human studies utilizing amino acids or metabolites/derivatives as a stand-alone intervention [58,59,60,61,62] or part of a multi-component strategy [56,57], seven were identified. There were six that utilized a controlled design [56,57,58,59,61,62] and one was a single-arm study [60]. There were six studies that measured body weight and muscle mass by bioelectrical impedance (BIA; one study also measured mid upper-arm muscle circumference “MAMC”); one study did not measure weight or muscle mass, but all studies measured PR-QOL. Among the studies, three assessed objective physical function by HGS, and three assessed subjective physical function. Additionally, three studies assessed all four outcome categories: body weight, muscularity, PR-QOL, and physical function [60,61,62]. Some improvements were reported for body weight [59], muscle mass [56,60], PR-QOL [58,59,60], and subjective physical function [58,60]; only one study reported an improvement in objective physical function (non-dominant HGS) [61].

#### 3.1.1. Essential Amino Acids

Leucine, isoleucine, and valine are essential branched-chain amino acids which may reduce proteolysis and enhance protein synthesis by activating the mTOR pathway (the primary anabolic pathway in skeletal muscle), and they may attenuate inflammation by increasing glutamine availability [80,81]. We identified five animal studies that administered leucine-rich diets for 2 to 3 weeks after tumor inoculation [19,22,23]. Leucine attenuated weight loss in three of these studies [22,23,24], and all five reported attenuated muscle wasting measured by select cross-sectional areas or muscle mass to body weight ratios. Only one assessed physical function and reported higher grip strength than tumor-bearing control [24]. A sixth leucine study initiated the intervention after animals had lost 5% body weight (12–15 days after tumor inoculation), and it was carried out until weight loss reached 20% (4–5 days) [20]. In that study, leucine, isoleucine, valine, or phosphate-buffered saline (PBS; control) was administered by oral gavage to mice. Leucine and valine each attenuated weight loss and increased gastrocnemius protein synthesis; leucine also increased soleus weight and reduced soleus protein degradation compared to the control [20]. No clinical studies were identified within our search parameters, although one recent study administered hypercaloric, hyperproteic leucine enriched oral supplements to cancer weight-losing patients, which resulted in the maintenance of body weight and an improvement of physical function, but it was no different than the control. It is important to note that the control arm was administered a standard hypercaloric, hyperproteic diet that also contained polyunsaturated fatty acids, had greater casein content, and less vitamin D [82].

#### 3.1.2. HMB, Arginine, Glutamine, and Glycine

Beta-hydroxy-β-methylbutyrate (HMB) is a metabolite of leucine which also upregulates the mTOR/p70 s6K pathway in muscle [20]. Glycine, another non-essential amino acid with anti-inflammatory properties, has displayed a benefit of muscle wasting on animal models, but the exact mechanism of its effect is unclear [83]. We identified three animal studies that assessed the effect of HMB or glycine in tumor-bearing animals. Male rats were given HMB-enriched (4%) or standard chow for 16 days prior to, and 1 week after, tumor inoculation [26]. Weight increased with HMB and decreased with standard chow compared to weight at tumor inoculation, and gastrocnemius weight (relative to body weight) increased with HMB compared to standard chow [26]. Male mice were treated with HMB (0.25 g/kg/d), EPA (0.6 g/kg/d), both, or PBS by oral gavage for 12 days, starting 9 days after tumor inoculation [25]. There was no effect of any treatment group on body weight, but all three treatments equally improved soleus mass and protein degradation compared to control [25]. Glycine did not attenuate weight loss, but it did attenuate muscle loss in some muscles, as well as improve latency to fall and grip strength, compared to the control after administration to male mice via subcutaneous injection for 3 weeks after tumor inoculation [27].

In humans, a meta-analysis of various patient cohorts with muscle atrophy reported that HMB, in combination with arginine (Arg) and glutamine (Gln), both non-essential amino acids, improved muscle mass, while HMB alone—or provided as a nutrient-dense oral nutritional supplement (ONS)—improved muscle strength, but it did not improve body weight [84]. In a recent systematic review of patients with active cancer (not specifically cancer cachexia), HMB supplementation, typically with Arg and Gln or in a nutrient-dense ONS, improved muscle mass in four out of four studies and physical function in two of two studies, but it did not improve PR-QOL or body weight [85]. However, determination of the benefits of HMB, Arg, and/or Gln, specifically in the setting of cancer cachexia, is still uncharacterized.

There were two controlled trials identified which administered HMB plus Arg and Gln, and they were compared to an isocaloric/isonitrogenous control in men and women with advanced solid tumors. Between these, one was a small study which reported that 4 weeks of HMB + Arg + Gln improved muscle mass, but the effect did not reach significance compared to control at 24 weeks; there was also no effect of time or treatment on body weight or PR-QOL [56]. A larger trial supplemented HMB + Arg + Gln (twice the dose used in that smaller study) for 8 weeks, and it did not observe a treatment effect on body weight, muscle mass (body plethysmography, BIA, or skin fold), or fatigue compared to control [57]. Neither study assessed physical function, but both studies reported low compliance in treatment and control groups, which suggests low feasibility. In addition, none of these studies reported quality assurance measures of the HMB formulations. A recent meta-analysis suggested that HMB is more beneficial for physical function when combined with exercise [86], but the impact of HMB, alone or in conjunction with exercise, on physical function in patients with cancer cachexia remains unclear.

#### 3.1.3. Carnitine

Carnitine is primarily obtained from foods such as red meat, fish, poultry, and lamb, but it is also synthesized from L-lysine and L-methionine in the kidney, liver, and brain. It transports fatty acids to the mitochondria for oxidation, which is an important source of energy in muscle [87,88], and circulating levels may be reduced in patients with cancer cachexia and malnutrition [89]. In two studies from the same laboratory, carnitine was administered, intragastrically, to male rats for 1 week after tumor inoculation [29,30]. Despite the same methodology and reported benefit on gastrocnemius and soleus in both studies, improved body weight was only observed in one report [30]. Physical activity, but not grip strength, also improved in one of those reports [29], but it was not measured in the other [30]. Similarly, gastrocnemius mass increased, with no effect on body weight, in a different study providing oral carnitine, for 1 week, to tumor-bearing male mice compared to control [28].

There were three small human trials that reported improvement in PR-QOL after supplementation with carnitine [58,59,60]. A pilot study in men and women with advanced cancer and carnitine deficiency reported improved fatigue, when controlling for baseline age and fatigue, and improved subjective physical function after 4 weeks compared to the control [58]. A double-blinded, randomized, controlled trial (RCT) in men and women with unresectable pancreatic tumors provided carnitine or placebo for 12 weeks, and it reported improved body weight and PR-QOL with carnitine compared to placebo with no difference in fatigue [59]. A single-arm study administered carnitine to men and women with advanced solid tumors for 4 weeks and reported increased lean body mass (LBM; BIA), as well as improved PR-QOL, with no change in body weight or HGS [60]. Quality assurances of the formulations were not explicitly reported; however, two of these three studies assessed circulating carnitine (both reported increased circulating levels) as a pseudo quality assurance measure [58,59]. It is reasonable to infer that carnitine was well-tolerated and displayed potential for improving PR-QOL. Larger studies are warranted to confirm its impact on QOL and explore its effect on clinical outcomes, such as physical function and body weight, with longer treatment duration.

#### 3.1.4. Creatine

Creatine is an organic compound produced in the liver and mainly stored in skeletal muscle as phosphocreatine, where it serves as an immediate fuel reserve for adenosine triphosphate regeneration during muscle contraction [90]. There were two animal studies that reported attenuation of weight loss and muscle wasting in tumor-bearing male rodents when administered via intraperitoneal (i.p.) injection [32] or in the drinking water [31]; improved grip strength was observed in one study compared to tumor-bearing controls [32].

Cancer cachexia may be associated with low circulating levels of creatine [32] and creatinine [91]; however, only two clinical trials were identified that examined creatine efficacy in cancer cachexia. A small double-blinded RCT tested creatine supplementation in men and women with advanced colorectal cancer undergoing chemotherapy; however, cachexia was not an explicit inclusion criterion [61]. After 8 weeks of creatine, body weight, muscle mass, physical function, or PR-QOL did not improve compared to placebo; however, a sub-group analysis suggested that creatine may be more efficacious for preserving body mass in those receiving milder chemotherapy [61]. A large double-blinded RCT provided creatine or placebo to men and women with incurable malignancy and cachexia/anorexia (≥5 lb. weight loss in the prior 2 months and/or estimated caloric intake < 20 kcals/kg/d, with the perception of loss of weight/appetite as a problem and weight gain as beneficial) [62]. There was no time or treatment effect on body weight, muscle mass (BIA assessed in a small sub-set), PR-QOL, or HGS after 1 month [62]. Creatine was generally well-tolerated, with the most frequent side effects being related to GI symptoms or shortness of breath. None of these studies reported any quality assurances of the creatine formulations.

While these well-designed creatine supplementation trials do not support a benefit on these outcomes, creatine, similar to HMB, is reportedly more efficacious when combined with resistance exercise in healthy and/or sarcopenic elderly patients [92]. In addition, improved muscle mass from other amino acids, particularly leucine, in combination with exercise, was observed in sarcopenic elderly men and women [93]; however, there is still no clear evidence to date, regarding the additive effect of amino acid supplementation with exercise, on the physical function or PR-QOL. Future trials should assess the impact of amino acid supplementation, alone and in combination with physical activity, on PR-QOL and physical function in patients with cancer cachexia.

### 3.2. Polyunsaturated Fatty Acids (PUFA)

Omega (ꞷ)-3 PUFAs are a group of long-chain fatty acids, including alpha-linolenic acid, EPA, and DHA; EPA and DHA are mainly found in fish oil and are the two most commonly evaluated PUFAs. They are thought to exert anti-inflammatory and antioxidant properties that may attenuate muscle wasting in cancer cachexia, though the exact mechanism remains unknown [94,95]. We identified five animal studies that administered PUFAs alone [33,34,35], or in combination with antioxidants [36,37], to tumor-bearing mice via an enriched diet. All five studies assessed body weight and muscle mass, with three reporting benefits on the outcomes [35,36,37]; two of the studies that administered PUFAs alone reported no benefit on weight or muscle mass [33,34]. Only one of these studies assessed physical function and reported greater handgrip strength [35].

Among human studies utilizing PUFAs alone [63,64,66,67,68,69,70,71,73] or in combination with antioxidants [74,75,76], 12 were identified. There were six that utilized a controlled design [68,70,71,74,75,76], three compared different experimental groups [67,69,73], and three were single-arm studies [63,64,66]. All studies assessed body weight, and 10 assessed PR-QOL. Muscularity was assessed by MAMC in three reports, by BIA in five reports (one also measured dual-energy x-ray absorptiometry), and by an unidentified method in one study. Objective physical function was measured by HGS in two studies, and subjective physical function was measured in six studies. There were five studies that assessed all four outcome categories: body weight, muscularity, PR-QOL, and physical function [68,69,70,71,75]. Some improvements were reported for body weight [63,64,66], with very few reports of improved PR-QOL [69,76], or subjective physical function [69,74].

#### 3.2.1. PUFAs Alone

The animal studies examining PUFAs in isolation administered either an ꞷ-3-FA-enriched diet or a control diet with a greater proportion of ꞷ-6-FA [33,34,35]. There was one study that primarily administered the diet prior to tumor inoculation [33], while the other had a longer treatment duration after tumor implantation [34]; nevertheless, both reported no effect on body weight or muscle mass. Wu et al. administered the diet after tumor inoculation, and they reported an improvement in handgrip strength, muscle mass, and body weight [35]. We identified nine human studies examining PUFAs alone. In a large double-blind RCT, EPA or placebo (medium chain triglyceride oil) were administered, for 2 months, to men and women with lung or gastrointestinal cancer and ≥5% loss of pre-illness weight [68]. A trend was observed for a difference in weight change (relative to placebo) between 2 g/d (+1.2 kg) and 4 g/d (+0.3 kg) after 2 months with no effect on LBM by BIA or PR-QOL. There was an improvement in subjective physical function with EPA 2 g/d compared to the other groups, with no differences in compliance or adverse event reporting [68]. Despite the fact that objective physical function was not measured, this is the only large clinical trial to assess PUFAs alone in the setting of cancer cachexia, and these results suggest that examination of EPA for improving physical function is still warranted.

There were two smaller RCTs that measured objective physical function after 7 weeks of PUFA administration during active anti-cancer treatment. In a phase II/III RCT, men and women with head and neck cancer ingested ꞷ-3-FA-containing echium oil or sunflower oil (without ꞷ-3-FA) [70]. There was no treatment effect on body weight, PR-QOL, or HGS over the course of the study; muscle mass was only assessed at 4 weeks, but FFM and LBM, assessed by dual-energy x-ray absorptiometry, decreased similarly in both groups with no change by BIA at that time point [70]. The lack of efficacy here may be explained by only one-third of patients experiencing weight loss history (≥5% over the prior 6 months) at enrollment, so patients may not have been cachectic as a group. Another small study administered fish oil or rapeseed oil to men and women with advanced lung cancer [71]. This study did not explicitly enroll cachectic patients either, and no treatment effect was observed for MAMC, PR-QOL, or HGS. While the fish oil group was weight stable at baseline (3-month history), the rapeseed oil group displayed significantly greater baseline weight loss (6.7%), which may suggest that rapeseed oil confers a weight-stabilizing effect considering the baseline weight loss displayed in that group [71]. Both studies confirmed that red blood cell ꞷ-3-FA content was increased after ꞷ-3-FA administration, suggesting that PUFAs may not be effective at improving weight, muscle mass, PR-QOL, or HGS in patients with advanced cancer and moderate or likely cachexia.

Small, multi-arm uncontrolled studies consistently reported a lack of effect on body weight or muscle mass when comparing fish oil to other supplements. Men and women with advanced gastrointestinal cancer and >10% weight loss over the prior 6 months consumed fish oil or melatonin for 1 month and, then, consumed both for 1 additional month; all patients received diet counseling at the baseline [67]. No group difference in weight or subjective physical function were detected at the end of the study despite confirmation of increased systemic ꞷ-3-FA [67]. Another study compared fish oil to a Chinese herb Atractylenolide I for 7 weeks in patients (sex unspecified) with advanced, unresectable gastric cancer and diminished or absent appetite (parameters undefined) [69]. No group differences were reported in rate of weight or MAMC change, but a greater rate of Karnofsky Performance Score increase was observed for Atractylenolide than fish oil [69]. These data may indicate potential for improved subjective physical function with this supplement; however, the potential for improved objective physical function remains untested. Men and women with pancreatic cancer and ≥5% weight loss since diagnosis were given fish oil in marine phospholipid form, which are purported to induce less of the classical gastrointestinal side effects attributed to the standard triglyceride form of fish oil [72,73]. Marine phospholipids or fish oil was administered for 6 weeks, but no change in body weight, muscle mass (method unclear), or PR-QOL was observed for either group [73]. While compliance was similar, less gastrointestinal side effects were reported with marine phospholipids, which may support further examination of this ꞷ-3-FA preparation, which displayed a similar weight-stabilizing effect to standard fish oil in that report [73].

In two small single-arm studies carried out by the same laboratory, the rate of weight change significantly improved after 3 months of fish oil or EPA administration to patients with unresectable pancreatic cancer [63,64]. No change was detected in MAMC and PR-QOL in the fish oil study [63], nor was there a change in subjective physical function in the EPA study [64], despite confirmation of increased systemic ꞷ-3-FA in both reports. Another single-arm study administered fish oil to men and women with advanced cancer and >2% weight loss over the prior month, and it reported that the number of days receiving fish oil was correlated with weight gain for those taking capsules over 30 days, but no change was observed in PR-QOL over the median treatment time of 1.2 months [66].

#### 3.2.2. PUFAs with Antioxidants

There were two animal studies that treated male tumor-bearing mice with PUFAs along with antioxidants, from 3 to 6 weeks, beginning the same day as tumor inoculation. Wang et al. provided chow enriched with fish oil, selenium, or both and found that only the combination improved body weight and muscle mass [37]. Similarly, van Norren et al. compared fish oil, leucine, or a high protein diet, individually or in combination, and found that only the combined intervention benefited body weight and muscle mass compared to the tumor-bearing control [36].

Among clinical studies, three assessed the impact of fish oil in combination with antioxidants [74,75,76]. Men and women with solid tumors, categorized as nourished or malnourished instead of by cachexia status, were provided with fish oil plus vitamin E or placebo, daily, for 40 days [74]. No change in body weight was observed in either group, but Karnofsky Performance Score increased in malnourished patients taking fish oil/vitamin E. In another study, men and women with advanced cancer, anorexia, and >5% weight loss from pre-illness weight were provided with fish oil, as well as vitamin E or olive oil, and they were instructed to take as many pills as tolerated (minimum 6 but not exceeding 18) daily for 2 weeks [75]. No group differences were reported for changes in weight, FFM (BIA), or PR-QOL in that study. Similarly, no group differences were observed in body weight, muscle mass, or PR-QOL after 2 months of protein supplementation, with or without EPA plus antioxidants, in men and women with pancreatic cancer and >5% weight loss over the prior 6 months, despite gas chromatography-confirmed elevation in phospholipids [76]. In that study, supplement intake was associated with improved body weight, lean mass (BIA), and PR-QOL for the protein/EPA/antioxidant group only, which may suggest an additive effect of EPA plus antioxidants with protein supplementation [76].

### 3.3. Polyphenols

Polyphenols contain several hydroxyl groups on aromatic rings, and they are classified by the number of rings present (stilbenes, flavonoids, lignans, or phenolic acids). They are available in many dietary sources, such as vegetables, fruits, coffee, tea, and wine, and their intake, especially flavonoids, has been associated with reduced incidence of several chronic diseases [96]. Polyphenol efficacy in the setting of cancer has been reviewed with inconclusive results, which is likely due to difficulty in evaluating dietary intake and the lack of large RCT that could provide more valid evidence [97].

We identified 13 animal studies that assessed different polyphenols in tumor-bearing mice. Of the studies, three studied the effect of quercetin through either gavage [38], enriched chow [39], or i.p. injection [40]. All assessed body weight and muscle mass, but only two measured physical function through grip strength, with one reporting an improvement [38]. On the other hand, we identified five other studies focusing on curcumin that assessed body weight and muscle mass, with four of them reporting a benefit in both [42,43,44,45]. Only two measured physical function by grip strength and reported a positive effect [44,45]. Additionally, two focused on silibinin and reported a benefit on muscle mass, body weight, and physical function [46,47]. An isoflavone diet was investigated in one study, which reported an improvement in only one muscle group [48]. There were three other studies that investigated resveratrol, assessing body weight and muscle mass, but only one measured physical function [44]. We only identified two human studies that focused on polyphenols and, specifically, curcumin. Both assessed muscle mass through BIA, physical function through HGS, and reported either body weight [78] or BMI [77]. Between them, one reported improvement in LBM and BMI [77]; however, neither study observed a significant impact on HGS.

#### 3.3.1. Quercetin

Quercetin is not synthesized in the human body, but it is one of the most readily available flavonoids with the highest abundance in onions, broccoli, apples, cherries, berries, tea, and red wine. It has been widely studied as an antioxidant and anti-inflammatory with health benefits, including decreased systolic blood pressure and low-density lipoprotein levels in overweight subjects at risk of heart disease [98]. In pre-clinical studies, quercetin has displayed promising chemo-therapeutic and chemo-preventive effects on many cancer types, but human studies are needed to confirm these potential benefits [99,100].

We identified three animal studies that administered quercetin to male mice with various colon cancer models. Velazquez et al. treated Apc^Min/+^ mice via gavage once animals displayed 1–4% weight loss (15 weeks of age) [38]. After 3 weeks, the quercetin-treated mice displayed less weight loss, as well as greater muscle mass and grip strength, compared to tumor-bearing control mice [38]. Levolger et al. reported similar results on body weight and muscle mass after administering quercetin-enriched chow for 3 weeks post-tumor inoculation to 8-week-old mice, but no effect was found on grip strength compared to control mice [39]. The lack of effect on grip strength in that study might be due to the intervention being initiated prior to cachexia development. VanderVeen et al. provided i.p. quercetin and chemotherapy for 5 days, starting 10 days after tumor inoculation, and observed an improvement in select muscle groups, but there was no body weight effect compared to control mice [40]. It is unknown whether an effect on body weight or overall muscle mass would be detected with extended quercetin treatment in that study. These animal data indicate a potential benefit of quercetin on muscle mass, particularly in colon cancer models of cachexia. No clinical studies administering quercetin to cancer patients with cachexia were identified within our search parameters, so the efficacy and safety of this intervention in cancer cachexia patients remains unknown, but further preclinical investigation could make quercetin a promising option for clinical testing.

#### 3.3.2. Curcumin

Similar to quercetin, curcumin is a flavonoid with widely studied antioxidant, anti-inflammatory, antimicrobial, and antitumor properties [101]. It is primarily found in turmeric, but it is known to display relatively low bioavailability in humans [102]. In the animal studies identified here, curcumin attenuated weight loss and muscle wasting compared to tumor-bearing control animals when treatment initiation was delayed after tumor inoculation. Additionally, four studies began curcumin administration after animals had developed weight loss and/or tumor palpability and were treated for 12–28 days [42,43,44,45]. Among these studies, two assessed physical function and reported greater grip strength with curcumin treatment compared to the tumor-bearing control [44,45]. There was one study identified that administered curcumin for 6 days, starting 1 day after tumor inoculation, and reported no effect on body weight or muscle mass [41].

In contrast to these promising preclinical observations, two double-blind phase II RCTs of curcumin supplementation were recently published, and they indicated good tolerance but low efficacy for improving cachexia outcomes after 8 weeks. In one report, curcumin or corn starch was administered to men and women with advanced solid tumors undergoing systemic anti-cancer treatment, but no time or treatment effects were reported for body weight, muscle mass by BIA, or HGS [78]. In a follow-up study, curcumin or a probiotic placebo was administered to patients (sex unreported) with advanced head and neck, or nasopharyngeal, cancer who were receiving palliative chemo or radiotherapy and were on a feeding tube [77]. Weight change was not reported, but there was no difference between groups in BMI change; however, muscle mass (BIA) increased with curcumin and decreased with probiotic control, with no time or treatment effect on HGS [77].

#### 3.3.3. Silibinin

Silibinin is a flavonoid found in milk thistle extract which has shown promising antioxidant, anti-inflammatory, and antitumor effects in lung [103], breast [104], colorectal [105], and non-melanoma skin [106] cancer models. There were two murine studies that administered silibinin 7 days after tumor inoculation, and they reported attenuation of tumor and/or chemotherapy-induced weight loss and muscle atrophy, as well as improved grip strength, compared to tumor-bearing control mice [46,47]. A single study administered silibinin, for 3 weeks, to female mice bearing pancreatic tumors [46], and the other study treated lung tumor-bearing male mice, intragastrically, for 8 days concomitantly with cisplatin [47]. In humans, silibinin improved brain edema in two patients with non-small cell lung cancer and brain metastases [107], but we did not identify any clinical trials evaluating its impact on cancer cachexia, which would be needed to assess its safety and efficacy. Further preclinical investigation could also make this a promising intervention strategy.

#### 3.3.4. Isoflavones

Isoflavones are a subclass of flavonoids found in soybeans that have shown the potential to decrease toxicity in cancer therapy [108]. We identified one study that administered an isoflavone diet to tumor-bearing male mice for 3 weeks post-tumor inoculation, and it reported improvement in the gastrocnemius mass, but there was no effect on body weight compared to tumor-bearing control [48].

#### 3.3.5. Resveratrol

Resveratrol is from the stilbene group of polyphenols and is abundant in grapes, berries, and red wine [109]. It is reportedly beneficial for cardiovascular health and exerts antioxidant, anti-inflammatory, and antitumor effects in multiple cell lines, but these effects have not been confirmed by clinical trials [110]. There were two animal studies that administered resveratrol to female mice (45–70 days old) for approximately 2 weeks, beginning 1–2 weeks after tumor inoculation [44,50]. Both studies observed attenuated weight loss and increased muscle mass compared to the tumor-bearing controls. Penedo-Vázquez et al. also assessed grip strength, as discussed with the curcumin studies, and found greater grip strength in the resveratrol-treated animals compared to tumor-bearing control mice; curcumin treated mice displayed greater grip strength than those treated with resveratrol [44]. Another study also administered resveratrol intragastrically to male mice similar to Shadfar et al. [50] but at much lower doses, and it reported no effect on body weight or muscle mass, even with the addition of fish oil [49].

### 3.4. Alkaloids

Alkaloids are a large group of chemical compounds that contain nitrogen, and their application has been studied in various diseases, including cancer, where some have been developed into chemotherapeutic agents. They are found in natural herbs, and they have promising effects as anticancer agents, but additional research and clinical trials are needed before recommendations for their use can be made [111,112]. We identified three animal studies that assessed alkaloids in male rodents. Zhang et al. administered matrine or sophocarpine i.p. to rats daily for 5 days, starting 12 days after inoculation with colon cancer cells, and they reported a prevention of weight and muscle loss with both interventions [52]. Iizuka et al. administered a Coptidis rhizome or berberine diet, and they reported the prevention of weight and muscle loss in mice bearing colon tumors [51]. Olivan et al. administered daily intragastric theophylline for 7 days, starting the same day as tumor inoculation, and reported an increase in the soleus mass but no difference in the other muscles studied or body weight between tumor-bearing groups [53]. Physical function was not assessed in any of these studies.

### 3.5. Probiotics

Probiotics are defined as “live microorganisms which when administered in adequate amounts confer a health benefit on the host” by the World Health Organization [113]. Their efficacy and safety have been reviewed in the setting of cancer with insufficient studies to draw any conclusions [114]. We identified two animal studies that assessed probiotics in mice bearing colon cancer. Varian et al. added Lactobacillus reuteri to the drinking water of ApcMin/+ mice for 3 months, and they found that treated mice presented with a greater muscle cross-sectional area and muscle:body weight ratio compared to controls. Kimchi is considered a probiotic meal that’s made by fermenting vegetables with probiotic lactic acid bacteria [115]. An et al. provided a kimchi diet to tumor-bearing male mice for 3 weeks, starting the same day as tumor inoculation, and observed that kimchi prevented weight loss and preserved muscle mass [55].

## 4. Discussion

There were 21 human and 37 animal studies identified within our pre-specified search criteria. In the animal studies, treatment efficacy on body weight and muscle mass were required for inclusion in this review; however, only 11 (29.7%) studies assessed physical function (mainly by grip strength). More than half the interventions primarily tested amino acids/derivatives or polyphenols. In the human trials, intervention efficacy was generally aimed at body weight, which was assessed in all but two studies (one of which measured BMI instead), and to a lesser extent, it was aimed at muscle mass and/or PR-QOL (measured in 71.4% of studies each). Subjective or objective physical function were assessed in only eight (38.1%) and six (28.6%) studies, respectively. Objective physical function was measured via HGS in these reports, except for one study that additionally reported knee extension and hip flexion strength, with overwhelmingly no significant time and/or group interactions. Interventions primarily utilized PUFAs (57.1%) or amino acids/derivatives (33.3%), and the well-powered, placebo-controlled trials in these categories revealed minimal evidence to support a benefit on body weight, muscle mass, or PR-QOL, with very few of these reporting any measure of physical function. Muscle mass was preserved in one of two curcumin RCTs, with no impact on HGS and no measure of PR-QOL in either one. These observations largely imply that, when administered outside the scope of complex multimodal interventions such as exercise and/or ONS, PUFAs and amino acids/derivatives do not confer significant benefits on body weight, muscle mass, or PR-QOL in the cancer cachexia setting. Although carnitine may have displayed some potential for improving PR-QOL, compared to placebo, in advanced-stage patients with weight loss or fatigue, larger, adequately powered studies are needed to confirm these findings. The only study to report improved objective physical function reported it for non-dominant HGS after creatine administration but not for placebo; however, a between-group comparison was not available for confirmation of the treatment effect [61].

Similarly, the American Society of Clinical Oncology and the European Society for Parenteral and Enteral Nutrition report low quality evidence and low benefit for PUFAs, vitamins, minerals, and other dietary supplements for body weight improvement, but they do consider PUFAs to be acceptable sources of dietary fats for patients with cancer cachexia [116,117]. There is not enough evidence to suggest whether PUFAs or amino acids/derivatives may benefit physical function in this setting because this outcome was largely untested in these cohorts. However, the European Society for Parenteral and Enteral Nutrition indicates that combined treatment with insulin and amino acids may benefit negative protein balance and anabolic resistance in cachexia [116], suggesting that further testing of the efficacy of amino acids/derivatives for improving physical function in this setting is warranted. In addition, of the amino acid/derivative studies in animals reviewed here, six focused on leucine supplementation, and they consistently reported improved muscle mass. Physical function was only assessed (and displayed improvement) in one of these studies, which may warrant further evaluation as an isolated modality in humans, considering that the two HMB human trials included in the present review did not assess physical function.

**Figure 1:**
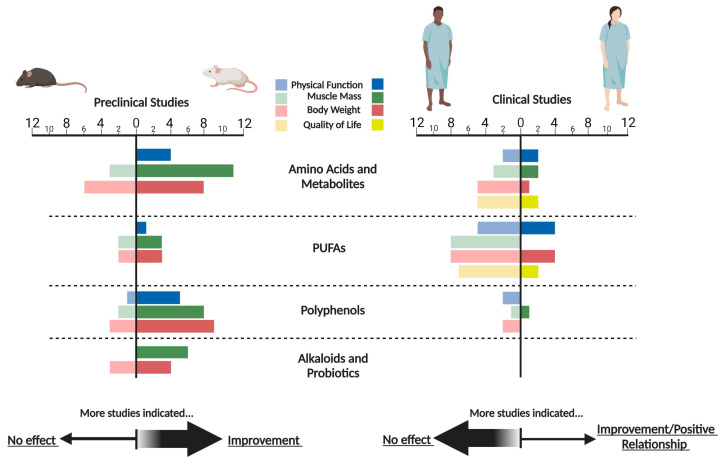
Frequency of studies reporting efficacy of nutraceuticals in cancer cachexia. Improvement/positive relationship includes any positive effects in the outcomes mentioned or positive correlations with the outcomes and the nutraceutical administered. Created with BioRender.com (accessed on 21 July 2023).

Overall, animal studies reported more favorable outcomes in muscle mass and body weight than clinical trials, and the few that assessed physical function were also more promising, as seen in Figure 1. This is not surprising, as this pattern has been seen in other interventions, such as ghrelin, where improvements in grip strength were reported in animal studies [118,119] but not in clinical trials utilizing anamorelin, a ghrelin receptor agonist [10].

The numerous inconsistencies observed here, across study designs, reflects the need for standardization of many parameters in this field of research. Some studies examined efficacy for cachexia amelioration and required various degrees of cachexia for study eligibility, while other studies investigated cachexia prevention and did not require cachexia at study entry but reported cachexia development using various definitions. There were eight (38.1%) human studies identified that were published after the consensus definition was established in 2011; however, the defining criteria for cachexia remains highly varied among clinical trials to date. We also observed a great deal of inconsistency across sample sizes, tumor types, and patient settings. For example, six (28.6%) studies required patients to be under/initiating active treatment, and five (23.8%) required patients to display anorexia or fatigue in addition to weight loss. There was also a large variation in muscle mass (predominantly assessed by BIA) and PR-QOL outcomes measures. In addition to the low abundance of functional outcomes, these limitations complicate the generalizability of findings to the larger cancer cachexia setting.

Intervention initiation in animal studies varied relative to tumor inoculation, with 22 (59.5%) studies beginning treatment prior to or along with tumor inoculation and 15 (40.5%) initiating the intervention around a week or more after tumor inoculation (delayed design). A delayed design may have the mosst clinical application, as it most closely resembles the typical human experience of tumor, and often cachexia, development prior to diagnosis and subsequent treatment. A cause for this discrepancy in design may be due to the lack of consensus definition for cachexia in animals. Another inconsistency was the age of the animals, which ranged from 3 to 18 weeks old. Age has been shown to aggravate cancer cachexia in a strain-dependent manner [120], and the translation of animal data to human patients is likely limited by the typical age of animals utilized in these studies, which generally reflect the human equivalent of adolescence or young adulthood and not the typical age of cancer diagnosis in human adults, which is closer to middle age [121].

## 5. Limitations

Many study design features contributed to variability in outcome efficacy and interpretation in this review. Treatment initiation seemed to divide the preclinical and clinical studies into two groups, with preclinical studies initiating treatment either at the time of tumor inoculation or after a certain degree of tumor-induced weight loss was achieved. In human trials, a similar situation occurred where patients were recruited based on the presence of cachexia (i.e., a certain degree of weight loss history) or based on likelihood of developing cachexia (i.e., patients with advanced cancer or those initiating systemic anti-cancer treatment). These are two different ways of assessing the efficacy of interventions in cancer cachexia, and they are almost always compared indifferently. The ages of the animals that were utilized also varied greatly among studies. Quality control of the nutraceutical formulations is needed to confirm that accurate doses are being administered; however, only a few clinical trials performed pseudo quality assurance by assessing changes in circulating levels of the nutraceutical. Several nutraceutical interventions were excluded from this review due to their combination with complex, multi-factorial designs, such as physical exercise or macronutrient-dense supplementation, and/or the absence of a proper control arm to appropriately evaluate efficacy. This confounds our ability to generalize our current findings to the potential additive effects of nutraceuticals on cachexia outcomes when combined with other interventions.

## 6. Conclusions 

Physical function, PR-QOL, and other clinically relevant endpoints should be the primary focus to evaluate intervention efficacy in cancer cachexia, yet as shown here, clinical studies primarily focus on body weight and, secondarily, on muscle mass and/or PR-QOL. The data synthesized here suggest that (1) the categories of nutraceuticals tested in humans, thus far, are either unlikely to benefit physical function, almost exclusively assessed via HGS, and/or (2) HGS is not the most suitable measure to capture the potential effect(s) on physical function. This review did not find supportive evidence that the nutraceuticals tested, so far, benefit physical function and QOL in cancer cachexia. However, amino acids/derivatives remain the most promising category of nutraceuticals for future examination. Considering the current barrier of improving physical function and QOL for therapeutic development, future clinical trials should investigate the efficacy of multimodal interventions on clinically relevant outcomes in the setting of cancer cachexia.

## Figures and Tables

**Table 1:** Preclinical data of nutraceutical interventions in tumor-bearing animals.

Strain	Tumor Model	Intervention ^A^	Duration	Outcomes of Interest	Ref
Amino Acids and Metabolites/Derivatives					
Amino Acids					
3/4-wk-old male Wistar rats.	Walker 256 (breast CA).	Control diet or High-Leu diet (HLD) (3%). Arms: CD; HLD; TB; TB + HLD (n = 8–10).	12 days.	Weight: No effect of HLD between TB groups. Muscle: Larger GSN in TB + HLD than TB. Physical Function: Not assessed.	[19]
NMRI mice (sex and age NR).	MAC 16 (colon CA).	PBS, Leu, IsoLeu, or valine (1 g/kg BW)/d by gavage. Initiated when mice lost 5% BW (12–15 days post-TI) Arms: TB; TB + Valine; TB + Leu; TB + IsoLeu. (n = 6).	4–5 days.	Weight: Leu & valine attenuated WL in TB. Muscle: Larger SOL with Leu in TB. Physical Function: Not assessed.	[20]
7–8 wks-old male CD2F1 mice.	C-26 (colon CA).	CD (8.7% Leu/g of PRO) or Leu chow [high (14.8% Leu/g of PRO) or low dose (9.6% Leu/g of PRO)]. Arms: CON; TB; TB + low; TB + high (n = 6).	21 days.	Weight: No effect of Leu. Muscle: Greater TA and GSN in TB + high than TB; no difference b/w TB + high or + low. Physical Function: Not assessed.	[21]
13-wk-old female Wistar rats.	Walker 256 (breast CA).	Isocaloric diets = CD (1.6% Leu) or HLD (3%). Arms: CD; HLD; TB; TB + HLD (TBL) (n = 6).	21 days.	Weight: Greater BW in TBL than TB. Muscle: No effect on muscle mass. Physical Function: Not assessed.	[22]
13-wk-old male Wistar rats.	Walker-256 (breast CA).	HLD (4.6%) or CD (1.6% Leu) Isocaloric for 21 days. Arms: CD; HLD; TB; TB + HLD (TBL) (n = 10).	21 days.	Weight: Greater weight gain in TBL than TB. Muscle: Greater GSN mass in TBL than TB. Physical Function: Not assessed.	[23]
12-wk-old Male Wistar rats.	Walker 256 (breast CA).	CD (18% PRO) or HLD diet: 18% PRO with 3% Leu ^B^ added. Arms: CD; HLD; TB; TB + HLD (TBL) (n = 6).	18 days.	Weight: TBL improved BW in TB. Muscle: Greater TA mass in TBL than TB. Physical Function: Greater HGS in TBL than TB.	[24]
ꞵ-hydroxy ꞵ-methylbutyrate/Glycine					
Male NMRI mice (age NR).	MAC16 (colon CA).	EPA (0.6 g/kg/d), HMB (0.25 g/kg/d), both, or olive oil or PBS (CON) by gavage. Initiated 9 days after TI. Arms: CON; EPA; HMB; HMB + EPA (n = 6).	9 days.	Weight: HMB attenuated WL in TB mice. Muscle: All had greater SOL mass than CON. Physical Function: Not assessed.	[25]
Male Wistar rats (age NR).	Yoshida ascites hepatoma.	4% HMB-enriched chow or standard chow (CD). Initiated 16 days before TI and continued for 8 days. Arms: CD; HMB; TB; TB + HMB (n = 12–15).	24 days.	Weight: HMB prevented WL in TB rats. Muscle: HMB attenuated GSN loss in TB. Physical Function: Not assessed.	[26]
14-wk-old male CD2F1 mice.	C-26 (colon CA).	1 g/kg/d of Glycine (Gly) or Saline in PBS via SC injections. Arms: CON + PBS; TB + PBS; TB + Gly (n = 12–16).	21 days.	Weight: No effect on BW. Muscle: Glycine attenuated wasting in TB. Physical Function: Greater latency to fall and HGS in TB + Gly than TB CON.	[27]
Carnitine					
7/9-wk-old male BALB/c.	C-26 (colon CA).	Oral L-CAR at 4.5 mg/kg/d or 18 mg/kg/d or saline (2 mL). Initiated 12 days after TI. Arms: CON; TB CON; TB + CAR (n = 5–8).	7 days.	Weight: No effect with CAR. Muscle: Greater GSN mass in TB + CAR than TB CON. Physical Function: Not assessed.	[28]
5-wk-old male Wistar Rats.	Yoshida ascites hepatoma.	Daily i.g. dose of CAR (1 g/kg of BW/d) or vehicle (corn oil). Arms: TB; TB + L-CAR (n = 8–24).	7 days.	Weight: Inconsistent BW effect. Muscle: TB + CAR reported inconsistent gains in some muscles than TB CO. Physical Function: Activity (not HGS) improved (2012); not measured in other (2020).	[29,30]
Creatinine					
Male Wistar rats (age NR).	Walker 256 (breast CA).	8 g/L of CRE monohydrate in drinking water (1.0 ± 0.1 g/kg/d). Initiated 11 days before TI and maintained for 10 days after. Arms: CON; TB CON; TB + CRE (n = 10).	21 days.	Weight: CRE prevented WL in TB mice. Muscle: CRE attenuated CSA of SOL and EDL but had no effect on muscle weight. Physical Function: Not assessed.	[31]
7-wk-old male BABL/c.	C-26 (colon CA).	Daily i.p. injection of CRE (125 mM) in PBS for 7 days. Arms: CON; TB CON; TB + CRE (n = 6).	7 days.	Weight: CRE alleviated WL in TB mice. Muscle: CRE attenuated muscle wasting in TB (TA, GSN, EDL, and SOL weight). Physical Function: Greater HGS in TB + CRE than TB.	[32]
Polyunsaturated Fatty Acids					
*PUFAs* Alone					
10-wk-old BDIX rats.	DHD/K12 colon CA, PROb clone.	CD = 12% peanut oil + 3% rapeseed oil or Fish Oil (FO): 8% peanut oil + 2% rapeseed oil + 5% FO. Some were pair fed (PF) and the rest fed ad libitum. Initiated 6 wks before TI and continued for 11 days. Arms: CD; TB + CD; CD + PF; FO; TB + FO; FO + PF (n = 10–18).	53 days.	Weight: FO had no effect on BW in TB mice. Muscle: No effect with FO on BW. Physical Function: Not assessed.	[33]
Male Fischer 344 rats (age NR).	Ward (colon CA).	Walnut (4.5 ꞷ-6/ꞷ-3 ratio) or CON (23.3 ꞷ-6/ꞷ-3 ratio) diet for a variable duration ^C^. Pair feeding (PF) began on day 31. The walnut diet was initiated on day 0 or the day after TI (day 21). Arms: CON diet; CON + walnut crossover; walnut diet (n = 6). Subgroups of Non TB; Non TB + PF; and TB in all 3 groups.	49–70 days.	Weight: No effect on BW in TB groups. Muscle: No difference in GSN muscle mass between TB groups. Physical Function: Not assessed.	[34]
7-wk-old C57BL/6J mice.	LLC (lung CA).	400 mg/kg of EPA-PL (EPA- enriched phospholipids) in corn oil or corn oil alone (CON) via gavage once a day. Initiated 8 days after TI. Arms: CON; TB CON; TB + EPA-PL (n = 8).	20 days.	Weight: EPA partially rescued BW loss. Muscle: EPA alleviated muscle wasting in TB QUAD and GSN (not TA, SOL, or EDL). Physical Function: TB + EPA had greater HGS than TB CON.	[35]
*PUFAs with Antioxidants*					
7–8 wks old male CD2F1.	C-26 (colon CA).	Normal diet (AIN93-M) + 22 g of FO (6.9 g EPA and 3.1 g DHA), 16 g/kg/d Leu, and/or HPD (151 g casein/kg/d). Arms: CON; TB; TB + HPD + Leu; TB + FO; TB + FO + HPD; TB + FO + Leu + HPD (n = 10–40).	20 days.	Weight: TB + FO + Leu + HPD attenuated WL. Muscle: Only TB + FO + Leu + HPD attenuated muscle wasting (TA). Physical Function: Not assessed ^D^.	[36]
6–7 wks old male BALB/cByJ mice.	Line-1 lung CA.	20 mg FO (EPA and DHA) and/or 0.69 mg selenium yeast (SY) with standard diet. Arms: CON; TB; TB + FO; TB + SY; TB + FO + SY (n = 6–10).	42 days.	Weight: TB + FO + SY attenuated WL in TB groups. Muscle: Greater GSN mass in TB + FO + SY than TB-CON, TB + FO, and TB + SY. Physical Function: Not assessed.	[37]
Polyphenols					
Quercetin					
15/18-wk-old male C57BL or ApcMin/+.	Colon CA.	25 mg/kg/d of Q or vehicle (tang juice + water) via gavage. Treatment started when mice lost 1–4% BW. Arms: C57BL/6; C57BL/6 + Q; TB CON, TB + Q (n = 5–8).	21 days.	Weight: TB + Q had less relative WL. Muscle: Greater muscle mass (GSN not QUAD) in Q + TB than TB CON. Physical Function: Greater HGS in Q + TB than TB CON.	[38]
8-wk-old male CD2F1 F1 mice.	C-26 (colon CA).	Regular or Q-enriched (250 mg/kg) chow. Expected daily intake of 35 mg/kg). Arms: CD; TB + CD; TB + Q (n = 10).	21 days.	Weight: TB + Q mice gained BW whereas TB + CD lost; significant group difference. Muscle: TB + Q presented greater GSN muscle mass than TB + CD. Physical Function: Both TB groups increased HGS; no group difference.	[39]
14-wk-old male CD2F1 mice.	C-26 (colon CA).	Fluorouracil (5 FU) 30 mg/kg of lean mass via i.p. with daily Q in propylene glycol (50 mg/kg of BW) or vehicle (propylene glycol) via gavage. Initiated 10 days after TI. Arms: CON; TB CON; TB + 5 FU; TB + 5 FU + Q (n = 5).	5 days.	Weight: Q had no impact on BW when compared to TB + 5 FU ^E^. Muscle: Greater EDL (not GSN, SOL, TA, or Plant) muscle mass and CSA in Q + TB + 5 FU than TB + 5 FU. Physical Function: Not assessed.	[40]
Curcumin					
Male Wistar rats (age NR).	Yoshida ascites hepatoma.	Curcumin (Curc) 20 mg/kg/d or vehicle i.p. Initiated 1 day after TI. Arms: CON; CON + Curc; TB CON; TB + Curc (n = 6–10).	6 days.	Weight: TB + Curc gained less BW than TB. Muscle: No effect. Physical Function: Not assessed.	[41]
6–7-wks-old male athymic mice.	MAC16 (colon CA).	100 mg/kg/d or 250 mg/kg/d of Curc or vehicle orally. Initiated 10–12 days after TI (5–7% WL). Arms: CON; TB; TB + 100 mg/kg; TB + 250 mg/kg (n = 5).	21 days.	Weight: Both Curc doses improved BW. Muscle: Greater GSN mass in Curc-treated mice than TB CON. Physical Function: Not assessed.	[42]
6-wk-old male BALB/c mice.	C-26 (colon CA).	Daily i.p. injection of 200 mg/kg of curcumin or PBS. Initiated 9 days after TI. Arms: CON; CON + Curc; TB CON; TB + Curc (n = 12–13).	7 days.	Weight: Greater BW in TB + Curc than TB CON. Muscle: TB + Curc had greater GSN and TA muscle mass than TB CON. Physical Function: Not assessed.	[43]
10-wk-old female BALB/c.	LPO7 (lung CA).	1 mg/kg/d of curcumin, 20 mg/kg/d of resveratrol, or saline via i.p. Initiated 15 days after TI. Arms: TB CON; TB + Curc; TB + Resv (n = 10).	15 days.	Weight: WL attenuated in both interventions. Muscle: Greater mass (GSN and SOL) in TB + Curc or Resv than TB CON. Physical Function: Greater HGS in TB + Curc or Resv (higher in + Curc) than TB CON.	[44]
6-wk-old female. BALB/c mice.	4T1 (breast CA).	“0.2 mL Curcumin solution of 150 mg/dL” or equal amount of saline by gavage. Initiated 1 wk after TI. Arms: CON + Saline; TB + CON; TB + Curc (n = 8).	28 days.	Weight: Greater BW in TB + Curc vs. TB CON. Muscle: Greater GSN mass in TB + Curc than TB + saline. Physical Function: Greater HGS at 2 and 4 wks in TB + Curc than TB + Saline.	[45]
Silibinin					
6–8 wks-old athymic female mice.	Human pancreatic CA S2-013.	200 mg/kg/d silibinin (SLI) or solvent control (form of administration NR). Initiated 7 days after TI. Arms: CON; TB CON; TB + SLI (n = 8).	18 days.	Weight: SLI attenuated WL in TB mice. Muscle: Greater mass (GSN) in TB + SLI than TB CON. Physical Function: Greater HGS and latency to fall in TB + SLI than TB CON.	[46]
5-wk-old male C57BL/6.	LLC (lung CA).	Cisplatin (DDP) 4 mg/kg or saline i.p. across 7 days (4 injections) + i.g. 0.3% sodium carboxymethyl cellulose, silibinin (SLI) 40 mg/kg/d (low dose), 0 or 80 mg/kg/d (high dose). Initiated 7 days after TI. Arms: CON; TB CON^C^; TB + DDP; TB + DDP + SLI 40; TB + DDP + SLI 80 (n = 5).	8 days.	Weight: SLI attenuated DDP WL in TB mice. Muscle: Greater GSN and TA in SLI 40 and 80 than TB + DDP. Physical Function: SLI improved HGS in a dose-dependent manner in TB + DPP groups.	[47]
Isoflavones					
8-wk-old male C57BL/6 mice.	LLC (lung CA).	CD with or without Isoflavones (obtained from soya flavone). Arms: CD; CON + Isoflavone; TB; TB + Isoflavone (n = 5–6).	21 days.	Weight: No difference between TB groups. Muscle: TB + Isoflavone presented greater muscle mass than TB (GNS. Not TA, SOL, EDL). Physical Function: Not assessed.	[48]
Resveratrol (Resv)					
5-wk-old male Wistar rats and 12 wk old C57Bl/6 mice.	Yoshida ascites hepatoma or LLC (lung CA).	Resveratrol 1, 5, or 25 mg/kg of BW or 3 mg/kg + 1 mL of FO i.g. Arms: CON; CON + Resv; TB CON; TB + Resv +/− FO (n = NR).	7 days.	Weight: No effect with resveratrol, even in combination with FO. Muscle: No difference between groups. Physical Function: Not assessed.	[49]
8–10-wks-old female CD2F1 mice.	C-26 (colon CA).	Resveratrol (100–500 mg/kg/d) or control vehicle by gavage. Initiated on the 6th day of TI. Arms: CON; CON + Resv 100–500 mg/kg; TB CON; TB + Resv 100–500 mg/kg (n = 4–8).	11 days.	Weight: WL attenuated in TB + Resv 200 and 500 mg/kg vs. TB CON. Muscle: Greater LBM and QUAD in TB + 200 and 500 mg/kg vs. TB CON. Physical Function: Not assessed.	[50]
Alkaloids					
6-wk-old male BALB/c mice.	C-26/ clone 20 (colon CA).	Coptidis rhizoma (CR) 1 or 2% or berberine (BB) (0.1–0.4%) in standard diet. Began 4 days prior to TI. Arms: CON; TB + CR (1–2%); TB; TB + BB (0.1–0.4%) (n = 6–9).	18 days.	Weight: BB and CR prevented WL in TB ^F^. Muscle: BB and CR prevented muscle wasting in TB. Physical Function: Not assessed.	[51]
Male BALB/c mice (age NR).	C-26 (colon CA).	Matrine (M) (50 mg/kg/d) or sophocarpine (SPH) (50 mg/kg/d) in 0.2 mL of Saline i.p. Initiated 12 days after TI. Arms: CON + Saline; TB + Saline; TB + M; TB + SPH (n = 10).	5 days.	Weight: M and SPH attenuated WL in TB mice. Muscle: M and SPH attenuated muscle wasting in TB mice. Physical Function: Not assessed.	[52]
5-wk-old male Wistar rats.	Yoshida ascites hepatoma.	Daily i.g. dose of theophylline (TPH), 50 mg/kg BW dissolved in corn oil or corn oil alone. Arms: CON; TB; TB + TPH (n = 6).	7 days.	Weight: No effect in BW with TPH in TB. Muscle: TB + TPH resulted in greater SOL mass (not GSN or TA) than TB CON. Physical Function: Not assessed.	[53]
Probiotics					
8-wk-old (sex NR). ApcMin/+ mice.	Colon CA.	*L. reuteri* (3.5 × 10^5^ organisms/mouse/d) in drinking water, replaced 2×/wk. Initiated 8 wks of age. Arms: TB; TB + *L. reuteri* (n = 6).	15 wks.	Weight: Greater muscle to BW ratio in *L. reuteri* than TB CON. Muscle: Larger GSN CSA in *L. Reuteri.* Physical Function: Not assessed.	[54]
6-wk-old male BALB/c mice.	C-26 (colon CA).	Probiotic-enriched Kimchi-diet (5.1 mg/kg/d) or normal diet (100 g/wk). Pellets were changed weekly. Arms: CON; TB; TB + kimchi diet (n = 10).	21 days.	Weight: Kimchi diet attenuated TB WL. Muscle: TB kimchi- preserved leg mass. Physical Function: Not assessed.	[55]

^A^ The intervention was initiated the same day as tumor inoculation (TI), unless otherwise noted. ^B^ The addition of 3% leucine was followed by a 1% reduction in corn starch (38.7%), dextrin (12.2%), and sugar (9%). ^C^ The experiment lasted for 70 days. On day 0, animals began with the control or walnut diet. Additionally, one day after tumor inoculation (day 21), half the animals on control diet were changed to the walnut diet. ^D^ Physical function was only assessed in an experiment where a diet containing all nutraceuticals was administered, together, without evaluating each separately. ^E^ Tumor-bearing control mice died on day 13, while the other groups were sacrificed on day 16. ^F^ CR at 2% greatly reduced food intake, reducing WL even more than TB CON. Wk(s), week(s); Leu, leucine; HDL, High Leucine Diet; TI, Tumor Inoculation; CD, Control Diet; TB, Tumor bearing; CON, Control; GSN, Gastrocnemius; NMRI, Naval Medical Research Institute; NR, not reported; MAC, Murine Adenocarcinoma; PBS, Phosphate Buffered Saline; g, gram; kg, kilogram; BW, body weight; d, day; WL, Weight loss; SOL, Soleus; PRO, protein; TA, Tibialis Anterior; HGS, handgrip strength; EPA, Eicosapentaenoic acid; HMB, Hydroxymethylbutyrate; Gly, glycine; SC, Subcutaneous; CAR, Carnitine; i.g., Intragastric; CRE, Creatinine; CSA, cross-sectional area; EDL, Extensor Digitorum; i.p., intraperitoneal; CA, Cancer; FO, fish oil; DHA, Docosahexaenoic acid; HPD, High Protein Diet; mg, milligram; Apc, Adenomatous polyposis coli; Min, Multiple Intestinal Neoplasia; Q, Quercetin; PLAN, plantaris muscle; LLC, Lewis lung carcinoma; LBM, Lean Body Mass; QUAD, Quadriceps; L. reuteri, Lactobacillus reuteri.

**Table 2:** Prospective nutraceutical interventions in patients with cancer cachexia.

Purpose	Design	Intervention	Efficacy Outcomes	Ref
Amino Acids and Metabolites/Derivatives				
HMB				
To assess the efficacy of HMB + Arg + Gln in cancer cachexia.	* Cohort: stage IV solid tumors. Cachexia I/E: WL > 5% (time frame unspecified).	EXP: HMB (3 g/d), Arg (14 g/d), Gln (14 g/d) juice. CON: isocaloric (180 kcal/d), isonitrogenous (7.19 g N/d) with non-essential amino acids. Assessed every 4 wks for 24 wks. EXP (n = 16 M/9 F); CON (n = 19 M/5 F).	Body Weight: No effect by time or treatment in intent-to-treat analysis. Muscle Mass: FFM change (BIA) was greater at Wk-4 in EXP (+1.12 kg) vs. CON (−1.34 kg) with a trend at Wk-24: EXP (+1.60 kg) vs. CON (+0.48 kg). PR-QOL: No changes or group difference in SF-36 or FACT-G. Physical Function: Not measured.	[56]
To assess the efficacy of HMB + Arg + Gln on prevention of LBM loss in cancer cachexia.	* Cohort: stage III/IV solid or metastatic cancer of any initial stage. Cachexia I/E: 2–10% WL over prior 3 mos.	EXP: HMB (3 g), Arg (14 g), Gln (14 g); bid. CON: isonitrogenous, isocaloric mixture; bid. Assessed after 8 wks. EXP (n = 145 M/75 F); CON (n = 143 M/83 F).	Body Weight: No group difference in change. Muscle Mass: No group difference in LBM change by BIA, skin fold, or body plethysmography. PR-QOL: No group difference in Schwartz Fatigue score. Physical Function: Not measured.	[57]
Carnitine				
Determine the effect of carnitine on fatigue in cancer patients with carnitine deficiency.	* Cohort: advanced cancer with fatigue and carnitine deficiency. Cachexia I/E: none and did not report BW at Pre or BW change.	EXP: L-carnitine 1 g in 10 mL syrup. CON: syrup (formulation not provided). Blinded phase: 5 mL/d for 2 d then 5 mL bid for 2 d then 10 mL twice/d for 10 d (2 wks total). Open phase (2 wks): same syrup progression as blinded phase + 2 g L-carnitine bid. Assessed at 2 and 4 wks EXP (n = 9 M/8 F), CON (n = 4 M/8 F).	Body Weight: Not measured. Muscle Mass: Not measured. PR-QOL: No group difference in FACT-An or LASA change (significance of within-group change NR; however, after controlling for baseline age and fatigue, FACT-An fatigue improved in L-carnitine vs. CON) after blinded phase. Physical Function: No group difference in KPS or FACT-An Functional Well-being sub-category after blinded phase.	[58]
Determine the effect of carnitine treatment in patients with advanced pancreatic cancer.	* Cohort: unresectable adenocarcinoma of the pancreas. Cachexia I/E: none but 90% had WL >10% in prior 6 mos.	EXP: L-carnitine “oral formulation” 4 g/d. CON: described as “identically formulated”. Assessed after 12 wks (caloric content not provided). EXP (n = 20 M/18 F); CON (n = 23 M/11 F).	Body Weight: L-Carnitine gained weight vs. placebo. Muscle Mass: BIA was measured but only body cell mass and body fat were reported. PR-QOL: Global QOL and GI symptoms from EORTC QLQ-C30 improved in L-carnitine vs. CON; no difference between groups in BFI. Physical Function: Not measured.	[59]
Efficacy and safety of L-carnitine in advanced cancer.	Cohort: solid tumors undergoing anti-cancer treatment. Cachexia I/E: none, but patients had to display fatigue and/or elevated ROS.	L-carnitine: 6 g/d (2 g tid); (n = 2 M/10 F). Assessed at 2- and 4-wks.	Body Weight: No change. Muscle Mass: LBM (BIA) increased at 2- (~1.7 kg) and 4-wks (~2.4 kg) vs. baseline. PR-QOL: MFSI-SF QoL “General Scale”, QoL-OS (all sub-scales), and EQ5D_VAS_ improved at 4-wks vs. baseline. Physical Function: MFSI-SF QoL “Physical Scale” and QoL-OS “Physical Scale” improved at 4-wks; no change in HGS.	[60]
Creatine				
Evaluate the effect of creatine on muscle function and QOL in patients with CRC.	* Cohort: CRC Stage III/IV undergoing chemotherapy. Cachexia I/E: none, but cachexia was a key feature of the background (results state none had >10% WL at Baseline).	EXP: creatine monohydrate. CON: cellulose Loading phase (1 wk): 5 g qid. Maintenance phase (7 wks): 2.5 g bid. Assessed after 8 wks. EXP (n = 10 M/6 F); CON (n = 10 M/5 F).	Body Weight: Increased in CON only. Muscle Mass: No change in MAMC or body cell mass (BIA) for either group (did not report lean mass from BIA). PR-QOL: No change in EORTC QLQ-C30 for either group. Physical Function: HGS increased for non-dominant hand in EXP; no change for either group in knee ext or hip flex.	[61]
To test the efficacy of creatine as a supportive care strategy in patients with cancer cachexia.	* Cohort: incurable malignancy (except primary brain tumor). Cachexia I/E: WL ≥ 5 lb in 2 mos, and/or estimated caloric intake < 20 kcals/kg/d and weight perception ^A^.	EXP: creatine monohydrate. CON: “identical-appearing placebo” Loading phase (5 d): 20 g/d. Maintenance phase (indefinitely): 2 g/d. assessed after 1; median treatment was ~2 mos for each group). EXP (n = 83 M/51 F); CON (n = 80 M/59 F).	Body Weight: No change in either group. Muscle Mass: No change in BIA parameters for either group (assessed in small sub-set). PR-QOL: No change in FAACT or linear analog self-assessment for either group. Physical Function: No change in HGS for either group.	[62]
Polyunsaturated Fatty Acids				
PUFAs Alone				
Study the effect of fish oil in weight-losing pancreatic cancer patients.	Cohort: unresectable adenocarcinoma of the pancreas. Cachexia I/E: none.	Fish oil: 2 g/d increased weekly by 2 g to a max dose of 16 g/d. Assessed at 1-mo and 3-mos. EXP (n = 18); sex unspecified.	Body Weight: Weight gain of 0.3 kg/mo at 3-mos was significantly different vs. rate of change at baseline (−2.9 kg/mo). Muscle Mass: No change in MAMC. PR-QOL: Not measured. Physical Function: Not measured.	[63]
To evaluate the acceptability and effect of oral EPA in weight-losing cancer patients.	Cohort: pancreas or ampulla (unresectable). Cachexia I/E: none.	EPA: initially 1 g/d increased to 6 g/d over 1 st 4 wks, then 6 g/d for remaining 8 wks; (n = 12 M/14 F). Assessed at 4, 8, and 12 wks.	Body Weight: Baseline WL averaged 13%; rate of loss was reduced at 4–12 wks. Muscle Mass: Not measured. PR-QOL: Not measured. Physical Function: No change in WHO performance status.	[64]
Examine the efficacy of fish oil to slow weight loss and improve QOL in cancer cachexia.	Cohort: malignancy not amenable to curative treatment. Cachexia I/E: WL > 2% prior 1 mo.	Fish oil: started at 0.3 g/kg/d fish oil, reduced to 0.15 g/kg/d after 13 patients; (n = 29 M/14 F). Assessed variably over 4 mos, 2 mos minimum. Dose derived from Phase I study with similar outcomes [65].	Body Weight: Number of days receiving fish oil was correlated with weight gain for those taking the capsules for ≥30 d. Muscle Mass: Not measured. PR-QOL: No change in FAACT or FACT-G. Physical Function: Not measured.	[66]
Study the effects of fish oil and/or melatonin in cancer cachexia.	Cohort: metastatic or locally advanced GI cancer not amenable to curative or standard palliative treatment. Cachexia I/E: >10% WL in prior 6 mos.	Fish oil: 30 mL/d (EPA 4.9 g + DHA 3.2 g); 4 wks. Melatonin: 18 mg/d; 4 wks. Cross-over: After initial 4 wks of treatment, all patients consumed both supplements for an additional 4 wks (all received diet counseling) Fish Oil (n = 7 M/6 F), Melatonin (n = 7 M/4 F).	Body Weight: No group difference in weight change at Wk 4 or Wk 8. Muscle Mass: Not measured. PR-QOL: No group difference in EORTC QLQ-C30 Global QoL. Physical Function: Baseline EORTC QLQ-C30 physical function was lower in Melatonin and increased at Wk 4 for Fish Oil; no group difference in KPS change.	[67]
To assess the effects of EPA on weight and LBM in cancer cachexia.	* Cohort: GI or lung. Cachexia I/E: ≥5% loss of pre-illness stable weight.	EXP: EPA 1 g in diester oil (2 or 4 g EPA/d). CON: MCT 1 g/d in diester oil. Assessed after 8 wks 2 g/d EPA (n = 117 M/58 F); 4 g/d EPA (n = 115 M/57 F); CON (n = 123 M/48 F).	Body Weight: Trend for between-group difference in change (relative to CON) at wk 8: 2 g (+1.2 kg) vs. 4 g (+0.3 kg). Muscle Mass: No group difference in LBM (BIA) change. PR-QOL: No group difference in EORTC QLQ-C30 for appetite. Physical Function: Physical function (EORTC QLQ-C30) improved in EPA 2 g vs. others; no group difference in KPS.	[68]
To assess the effects of largehead atractylodes rhizome in alleviating cytokine-mediated symptoms in cancer cachexia.	Cohort: advanced, unresectable gastric cancer. Cachexia I/E: diminished or absent appetite (undefined).	EXP1: Atractylenolide I (1.32 g/d; 6 ml bid). EXP2: Fish Oil (0.45 g/d; 4 pills bid). 3 wks treatment, 1 wk rest, 3 more wks treatment; assessed after 7 wks. EXP1 (n = 11); EXP2 (n = 11); sex NR.	Body Weight: No group difference in rate of weight change. Muscle Mass: No group difference in rate of MAMC change. PR-QOL: Greater rate of VAS appetite increase at 3 and 7 wks in EXP1 vs. EXP2. Physical Function: Greater rate of KPS increase at 3 and 7 wks in EXP1 vs. EXP2.	[69]
To test the efficacy of echium oil as a supportive care strategy in HNC in systemic therapy.	* Cohort: HNC initiating radio-chemotherapy. Cachexia I/E: none but average WL was 2.4% at baseline and 30% had ≥5% 6-mo WL at baseline.	EXP: 7.5 mL echium oil (235 ± 30 mg/mL ALA + 95 ± 13 mg/mL ALA SDA + 79 ± 10 mg/mL GLA) bid. CON: 7.5 mL sunflower oil (no ꞷ-3-FA) bid. From therapy initiation, assessed after 7 wks. EXP (n = 36 M/7 F); CON (n = 35 M/7 F).	Body Weight: No group difference. Muscle Mass: No group difference in FFM and LBM (DXA) decrease; no change by BIA (DXA and BIA assessed at Wk 4). PR-QOL: EORTC QLQ-C30 and -H&N35; no within-group changes or between-group difference. Physical Function: no within-group changes or between-group difference in HGS change.	[70]
Assess if fish oil has beneficial effects on weight loss in lung cancer patients.	* Cohort: advanced lung cancer undergoing chemotherapy. Cachexia I/E: none.	EXP: “Fish Oil” EPA 0.1 g/mL + DHA 0.12 g/mL. CON: “Rapeseed Oil” ALA 0.078 g/mL. 60 mL/d (20 mL/meal/d); average treatment duration was 48–49 d. EXP (n = 13 M/7 F); CON (n = 9 M/13 F)	Body Weight: No WL in either group. Muscle Mass: No change in MAMC for either group. PR-QOL: No change in EORTC QLQ-C30 or Lung Cancer-13 for either group. Physical Function: No change in HGS for either group.	[71]
Compare MPL and fish oil on weight, appetite, and QOL in pancreatic cancer patients with cachexia.	Cohort: pancreas Cachexia I/E: WL ≥ 5% since diagnosis (Building on their prior pilot study [72]).	EXP1: “MPL” (35% ꞷ-3-FA phospholipids + 65% neutral lipids). EXP2: “Fish oil” (60% EPA/DHA + 40% MCT) Both have same ratio EPA:DHA; total ꞷ-3-FA dose 300 mg/d both groups. Assessed after 6 wks MPL (n = 9 M/6 F); Fish oil (n = 7 M/11 F).	Body Weight: No change in either group. Muscle Mass: “Muscle mass” (undefined) not different between groups at Wk 6. PR-QOL: EORTC QLQ-C30 (no change in either group), PAN26 (hepatic function improved in MPL only). Physical Function: Not measured.	[73]
PUFAs with Antioxidants				
Investigate the effect of PUFA’s on T-cell subsets and cytokine production in cancer patients with or without malnutrition.	* Cohort: solid tumors. Cachexia I/E: none but groups were divided into well-nourished and malnourished ^B^.	EXP: “Fish oil” EPA 170 mg, DHA 115 mg + Vitamin E 200 mg; 6 pills tid. CON: “sugar tablets”; 6 pills tid. Total Fish Oil 18 g/d; assessed after 40 days. EXP (n = 17 M/13 F); CON (n = 19 M/11 F).	Body Weight: No change in either group. Muscle Mass: Not measured. PR-QOL: Not measured. Physical Function: Increased KPS in malnourished EXP patients only.	[74]
Determine whether fish oil at high doses improves symptoms in advanced cancer patients with weight loss and anorexia.	* Cohort: advanced cancer. Cachexia I/E: anorexia (>3 on VAS) + >5% WL from pre-illness weight.	EXP: “Fish Oil” 1000 mg = EPA 180 mg, DHA 120 mg, and vitamin E 1 mg. CON: 1000 mg olive oil as many as tolerated, up to 18 pills/d for 2 wks EXP (n = 10 M/20 F); CON (n = 7 M/23 F).	Body Weight: No group difference in change. Muscle Mass: No group difference in FFM (BIA) change. PR-QOL: No group difference in VAS change for appetite, nausea, tiredness, or overall well-being. Physical Function: No group difference in KPS change.	[75]
Assess the effects of a fatty acid and antioxidant enriched supplement on weight, body composition, diet, and QOL in weight losing pancreatic cancer patients.	* Cohort: unresectable adenocarcinoma of the pancreas. Cachexia I/E: WL > 5% in prior 6 mos.	EXP: 16 g PRO, 6 g fat, 1.1 g. EPA and antioxidants [Vitamins A 2524 IU, E 75 IU, C 105 mg, and selenium 17.5 mg]); 2 cans/d (620 kcals/d). CON: 16 g PRO, 6 g fat; 2 cans/d (620 kcals/d) Assessed after 8 wks EXP (n = 54 M/41 F); CON (n = 56 M/49 F). Separate post-hoc analysis for compliant (≥1.5 cans/d) vs. non-compliant (<1.5 cand/d).	Body Weight: No group difference in change. Supplement intake correlated with weight gain in EXP; trend for weight gain over 8 wks in compliant vs. WL in non-compliant. Muscle Mass: No group difference in LBM (BIA) change. Supplement intake correlated with LBM gain in EXP. PR-QOL: Supplement intake correlated with EuroQol EQ5D_index_ increase in EXP; trend for better EORTC QLQ-C30 over 8 wks in compliant vs. non-compliant. Physical Function: Not measured.	[76]
Polyphenols				
Curcumin				
Determine the effect of curcumin in HNC cachexia.	* Cohort: HNC or nasopharyngeal receiving chemo- or radiotherapy + feeding tube. Cachexia I/E: >5% WL in prior 6 mos or 2–5% WL + BMI < 20 kg/m^2^.	EXP: Curcumin (2000 mg bid: 4 capsules of 500 mg each). CON: matching placebo “made from probiotics” (2000 mg bid: 4 capsules of 500 mg each). Assessed after 8 wks. EXP (n = 10); CON (n = 10); sex NR.	Body Weight: NR, but BMI change was not different between groups. Muscle Mass: LBM (BIA) change after 8 wks was significantly different between curcumin (+0.46 kg) and CON (−1.05 kg). PR-QOL: Not measured. Physical Function: No change in HGS for either group.	[77]
Evaluate the effect of curcumin on body composition in cancer cachexia.	* Cohort: advanced solid tumors, undergoing systemic treatment. Cachexia I/E: WL ≥ 5% in 12 mos or BMI < 20 kg/m^2^ + 3 criteria ^C^.	EXP: Curcumin (800 mg bid). CON: Corn starch (800 mg bid). Assessed after 8 wks (caloric content not provided). EXP (n = 12 M/5 F); CON (n = 14 M/2 F).	Body Weight: No within- or between-group differences. Muscle Mass: Skeletal muscle mass (BIA), no within- or between-group differences. PR-QOL: Not measured. Physical Function: No within- or between-group differences in HGS.	[78]

* Randomized controlled trial. Samples sizes reflect randomization/baseline numbers. Within-group and between-group differences or changes are statistically significant unless otherwise noted. ^A^ Patients had to perceive loss of weight/appetite as a problem, and physicians had to view weight gain as beneficial; ^B^ Well-nourished [<10% WL in prior 6 mos, serum albumin > 30 g/L, serum transferrin > 2.0 g/L, and KPS > 60] vs. malnourished [>10% WL in prior 6 mos, serum albumin < 30 g/L, serum transferrin < 2.0 g/L, and KPS < 60]; ^C^ Additional criteria: deceased muscle strength, fatigue, anorexia, low fat-free muscle index, or abnormal biochemistry (increased inflammatory markers: C-Reactive Protein/Interleukin-6; Anemia: hemoglobin < 12 g/dL; low albumin: <3.2 g/dL). ALA, α-linolenic acid (omega-3 PUFA); Arg, L-arginine; BFI, Brief Fatigue Inventory; BIA, bioelectrical impedance analysis; bid, twice a day; BMI, body mass index; BW, body weight; CON, control/placebo group; CRC, colorectal cancer; d, day; DHA, docosahexaenoic acid (omega-3 PUFA); DXA, dual-energy x-ray absorptiometry; EORTC QLQ-C30, European Organization for Research and Treatment of Cancer Quality of Life Questionnaire; EPA, eicosapentaenoic acid (omega-3 PUFA); EuroQol EQ5D_index_, EuroQol-5 Dimensions-3 Level; EXP: Experimental; F, females; FAACT, Functional Assessment of Anorexia/Cachexia Therapy; FACT-G/An, Functional Assessment of Cancer Therapy-General/Anemia; FFM, fat-free mass; GI, gastrointestinal; GLA, γ-linolenic acid (omega-6 PUFA); Gln, L-glutamine; HGS, handgrip strength; HMB, ß-hydroxy-ß-methylbutyrate; HNC, head and neck cancer; I/E, inclusion/exclusion; kcals, kilocalories; KPS, Karnofsky Performance Score; LASA, Linear Analogue Scale Assessments; LBM, lean body mass; M, males; MAMC, mid upper-arm muscle circumference; MCT, medium chain triglyceride; MFSI-SF QoL, Multidimensional Fatigue Symptom Inventory-Short Form; mos, months; MPL, Marine phospholipids; NR, not reported; NSCLC, non-small cell lung cancer; ꞷ-3-FA, omega-3-fatty acid(s); PAN26, QOL in pancreatic cancer patients; PR-QOL, patient-reported quality of life; PRO, protein; PUFA, polyunsaturated fatty acid; qid, four times a day; QoL-OS, quality of life related to oxidative stress; ROS, reactive oxygen species; SDA, stearidonic acid (omega-3 PUFA); SF-36, Short Form-36 Health Survey; tid, three times per day; VAS, visual analog scale; WHO, World Health Organization; wk(s), week(s); WL, weight loss.

## Data Availability

No new data were created or analyzed in this study. Data sharing is not applicable to this article.
